# SPIKY: A graphical user interface for tracking spike train similarity

**DOI:** 10.1186/1471-2202-15-S1-P201

**Published:** 2014-07-21

**Authors:** Thomas Kreuz, Nebojsa Bozanic

**Affiliations:** 1 Institute for Complex Systems, CNR, Sesto Fiorentino, Italy

## 

Modern electrophysiology is developing fast and it sometimes provides more data than available algorithms and methods can handle and process. In particular, there is an increasing demand for methods which provide the possibility to study similarity patterns of activity across many neurons. Accordingly, a wide variety of approaches to quantify the similarity (or dissimilarity) between two or more spike trains has been suggested. Recently, the ISI- and the SPIKE-distance [1,2] have been proposed as parameter-free and time-scale independent measures of spike train synchrony. The key property of both measures is that they are time-resolved since they rely on instantaneous estimates of spike train dissimilarity. This makes it possible to track changes in instantaneous clustering, i.e., time-localized patterns of (dis)similarity among multiple spike trains. The SPIKE-distance also comes in a causal variant [2] which is defined such that the instantaneous values of dissimilarity are defined from past information only so that time-resolved spike train synchrony can be estimated in real-time.

For both the regular and the real-time SPIKE-distance, there are several levels of information reduction [3]. The starting point is the most detailed representation in which one instantaneous value is obtained for each pair of spike trains. This results in a matrix of size ’number of sampled time instants’ × ’squared number of spike trains’ (i.e. #(t_n_)N^2^). By selecting a pair of spike trains one obtains a bivariate dissimilarity profile whereas the selection of a time instant yields an instantaneous matrix of pairwise spike train dissimilarities which can be used to divide the spike trains into instantaneous clusters, i.e., groups of spike trains with low intra-group and high inter-group dissimilarity. Another way to reduce the information is averaging. The spatial average over spike train pairs yields a dissimilarity profile for the selected (sub)population, whereas temporal averaging leads to a bivariate distance matrix for the selected interval or the selected trigger points. Finally, application of the remaining average results in one distance value which describes the overall level of synchrony for a group of spike trains over a given time interval.

The Matlab source codes for calculating and visualizing both the ISI- and the SPIKE-distance have been made publicly available and have already been widely used in various contexts. However, the use of these codes is not very intuitive and their application requires some basic knowledge of Matlab. Thus it became desirable to provide a more user-friendly and accessible interface. Here we address this need and present an advanced version of the graphical user interface SPIKY [4,5]**.** This interactive program facilitates the application of the ISI- and the SPIKE-distance to both simulated and real data. Once given a set of spike train data (importable from many different formats), SPIKY calculates the desired measure and allows visualization of different representations such as measure profiles and pairwise dissimilarity matrices. SPIKY also includes the possibility to generate movies which are very useful in order to track the varying patterns of (dis)similarity. Finally, SPIKY has been optimized with respect to both computation speed (by using MEX-files, i.e. C-based Matlab executables) and memory demand (by taking advantage of the piecewise linear nature of the dissimilarity profiles).
